# Troubles de l'hémostase en réanimation chirurgicale de l'Hôpital Universitaire Joseph Ravoahangy Andrianavalona Antananarivo

**DOI:** 10.11604/pamj.2015.22.114.6220

**Published:** 2015-10-12

**Authors:** Lalaina Elianah Rasoamampianina, Toky Andriamahefa Rafanomezantsoa, Aurélia Rakotondrainibe, Andriambelo Tovohery Rajaonera, Zely Arivelo Randriamanantany, Olivat Rakoto Alson, Nasolotsiry Enintsoa Raveloson

**Affiliations:** 1 USFR Réanimation Chirurgicale HJRA, Faculté de Médecine Université Antananarivo, Madagascar; 2 UPFR Immunologie, HJRA, Madagascar; 3 UPFR Hématologie Biologique HJRA, Faculté de Médecine Université Antananarivo, Madagascar; 4 USFR Urgences et Réanimation Médicale HJRB, Faculté de Médecine Université Antananarivo, Madagascar

**Keywords:** Coagulation, réanimation, transfusion, trouble de l′hémostase, Coagulation, resuscitation, transfusion, hemostasis disorder

## Abstract

Les troubles de l'hémostase sont fréquemment observés en réanimation. L'objectif de cette étude est d'en déterminer l'incidence, les étiologies et la prise en charge pour en diminuer le risque de morbi-mortalité. Etude rétrospective sur une période de huit mois en réanimation chirurgicale du CHUA HUJRA. Les dossiers étudiés étaient ceux contenant tous les premiers bilans d'hémostase lors de l'admission du patient en réanimation, avec un âge supérieur à 15 ans. Etaient exclus les dossiers des post opérés (malade ayant subi une intervention chirurgicale). Les dossiers retenus pour l'étude étaient ceux qui ont présenté une ou plusieurs anomalies au niveau du bilan d'hémostase. Cent cinquante-cinq dossiers ont été colligés dont 20,64% ont présenté un trouble de l'hémostase. Les patients de sexe féminin (53,12%) étaient les plus concernés, avec une population âgée en moyenne de 49±19 ans. Trois patients (9,37%) ont présenté une notion de diathèse hémorragique à l'interrogatoire. Les différents troubles de l'hémostase étaient: une diminution du taux de prothrombine (100%), un taux de céphaline activé allongé (78,12%), un international normalized ratio élevé (43,75%) et des thrombopénies (40,62%). Chez 3,12% de ces cas, des accidents hémorragiques ont survenu. La plupart de ces troubles de l'hémostase étaient présents au décours d'hémorragies digestives (46,87%) dont la prise en charge était constituée par l'administration de vitamine K1 (68,75%) et de transfusion de plasma frais congelé (65,62%). D'autres pathologies ont été également incriminées. Une incidence de 15,62% de mortalité a été retrouvée. L'existence des troubles de l'hémostase en réanimation constitue un facteur pronostique imposant un dépistage et une prise en charge précoces et adéquats pour pouvoir en réduire le taux de mortalité.

## Introduction

Les troubles de l'hémostase sont fréquemment observés en réanimation [1]. L'hémostase résulte des interactions existantes entre la paroi vasculaire, les plaquettes et les facteurs de coagulation [2]. Les objectifs de notre étude étaient de déterminer la prévalence et la prise en charge ainsi que l'évolution de ces troubles.

## Méthodes

Il s'agit d'une étude rétrospective sur 9 mois allant de mars à décembre 2013, par consultation des dossiers médicaux des patients admis en Réanimation chirurgicale à l'Hôpital Universitaire Joseph Ravoahangy Andrianavalona Antananarivo. Les patients inclus dans cette étude ont un âge supérieur ou égal à 15 ans, avec bilan d'hémostase dès l'admission (TP, TCA, INR, numération plaquettaire) et un dossier médical exploitable. Ont été exclus les post- opérés et les dossiers incomplets. Les paramètres analysés étaient l'âge, le genre, la classification ASA du patient, le motif d'admission, les résultats du bilan de l'hémostase, la prise en charge et l'évolution. Les données ont été enregistrées sur la base de registre du logiciel Excel de Microsoft et l'analyse statistique faite par le test de corrélation de Pearson (SigmaStat^®^3.5). Une valeur de p inférieure à 0,05 a été retenue significative. Nos résultats sont exprimés en moyenne ± écart-type ou avec les extrêmes et en pourcentage.

## Résultats

**Figure 1:**
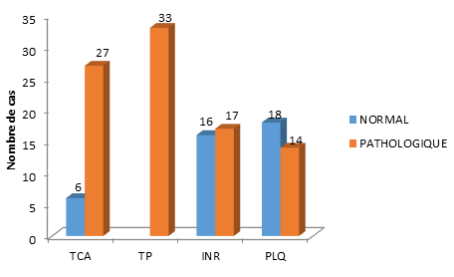
Répartition des patients en fonction du trouble de l'hémostase

**Figure 2:**
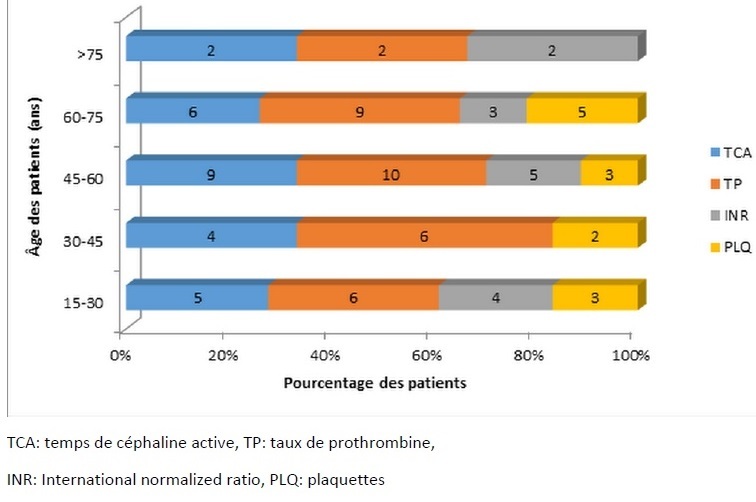
Relation entre la survenue des troubles de l'hémostase et l’âge des patients

Durant la période d'étude (9 mois), 155 dossiers médicaux ont été retenus avec 20,64% de troubles de l'hémostase retrouvés (thrombopénie, TCA allongé, baisse du TP, INR élevé) sans antécédent de prise médicamenteuse altérant l'hémostase. Sur le profil épidémiologique, on a une prédominance féminine à 53,12%. L'âge moyen est de 49±19 (16 à 84) ans. Les patients classés ASA 1 représentaient 81,25%. Trois patients (9,37%) ont présenté une notion de diathèse hémorragique à l'interrogatoire. Les différents troubles de l'hémostase (Figure 1) étaient: une diminution du taux de prothrombine (100%), un taux de céphaline activé allongé (78,12%), un international normalized ratio élevé (43,75%) et des thrombopénies (40,62%). Chez 3,12% de ces cas, des accidents hémorragiques sont survenus. La survenue d'un trouble de l'hémostase n'est pas significativement en relation avec l'âge. (Figure 2). Elle concerne également les deux genres. La plupart de ces troubles de l'hémostase étaient présents au décours d'hémorragies digestives (46,87%), un traumatisme crânien ou un polytraumatisme (7 patients), et autres pathologies pour 10 patients (pathologies tumorales, défaillances organiques). La survenue d'une thrombopénie est associée avec les hémorragies digestives et dans le cadre des traumatismes crâniens avec des valeurs significatives de p à 0,05 et 0,009. La prise en charge dans notre service était constituée par l'administration de vitamine K1 (68,75%), d'une transfusion de plasma frais congelé (65,62%) et une administration intraveineuse d'acide tranexamique et de l'etamsylate (9 patients). Une incidence de 15,62% de mortalité a été retrouvée.

## Discussion

Les troubles de l'hémostase sont des réalités fréquentes en réanimation. Les prévalences retrouvées par d'autres études sont assez variables: 20,64% dans notre étude contre 10,5% à Casablanca [3], 48% en Italie [4], et 16% au Royaume Uni [5]. L'âge moyen de survenue de ces troubles est aux environs de la quarantaine identiquement à celle retrouvée dans la littérature [3–5]. Notre étude a vu une prédominance féminine à 53,12% alors que les auteurs ont retrouvé une fréquence des troubles de l'hémostase chez le genre masculin: 68% en Italie, 65% au Royaume Uni, 59% à Casablanca [3–5]. Concernant les troubles de coagulation fréquemment retrouvés en réanimation, les thrombopénies sont à la première place. Elles peuvent être présentes dès l'admission ou acquise [6, 7]. Au Royaume uni, 16,4% des patients ont présenté des hémorragies digestives. Les insuffisances hépatocellulaires dans 70% des cas sont associées à une baisse du taux de prothrombine, un allongement du TCA, un INR élevé et une diminution du taux des plaquettes. La fibrinogénémie est à la limite de la normale [5]. Sur la prise en charge, dans notre étude, la vitamine K1, le plasma frais congelé associés plus ou moins à l'etamsylate ou l'acide tranexamique sont les bases du traitement.

Selon les auteurs, la prise en charge varie en fonction de la nature du trouble de l'hémostase rencontré. Une transfusion plaquettaire est réalisée pour maintenir un chiffre de plaquettes supérieur à 10.10^9^. L^-1^ [8]. Au Royaume uni, 72% des patient présentant une hémorragie digestive sont traités par culot globulaire, 36% par du PFC [5]. Les patients thrombopéniques sont les récipiendaires de la majeure partie des produits sanguins labiles délivrés dans les unités de réanimation [9, 10]. Pour les INR élevés, la prise en charge consiste à une administration de plasma frais mais pour les cas d'intolérance au plasma, le relais par un concentré de complexe prothrombinique est recommandé [11]. Une baisse du taux de prothrombine doit être corrigée selon le degré de gravité, par l'administration de vitamine K1: pour une correction rapide la voie IV est de mise mais pour les corrections non-urgentes on prescrit la voie per os. En réanimation l'administration sous 'cutanée est préférable [12, 13]. La mortalité associée aux troubles de l'hémostase en réanimation est encore faible dans cette étude, elle est de 48% à Casablanca [3]. Une numération plaquettaire basse dès l'admission ou acquise rapportent une surmortalité en réanimation, la thrombopénie est un facteur indépendant de mortalité qui multiplie par 4,2 [14]. Notre étude a été limitée par la taille de l'échantillon, l'absence de l'exploration complète de l'hémostase telle la fibrinogénémie, recommandée devant les troubles hémorragiques [15]. La baisse du taux de fibrinogène est associée à une majoration du risque hémorragique [16]. Sur le plan thérapeutique, nous avons été limités par le pouvoir des patients et la disponibilité des produits sanguins labiles.

## Conclusion

Les troubles de l'hémostase compliquent souvent la prise en charge des patients en réanimation. Une bonne évaluation clinique et des examens hématologiques bien conduits permettront une prise en charge adéquate compromettant l'évolution du patient réduisant ainsi la morbi-mortalité.
